# Predictors of surgical site infections among patients undergoing major surgery at Bugando Medical Centre in Northwestern Tanzania

**DOI:** 10.1186/1471-2482-11-21

**Published:** 2011-08-31

**Authors:** Brian Mawalla, Stephen E Mshana, Phillipo L Chalya, Can Imirzalioglu, William Mahalu

**Affiliations:** 1 Department of Surgery Weill Bugando University College of Health Sciences, Mwanza, Tanzania; 2 Department of Microbiology/Immunology Weill Bugando University College of Health Sciences, Mwanza, Tanzania; 3 Institute of Medical Microbiology, Giessen, Germany

## Abstract

**Background:**

Surgical site infection (SSI) continues to be a major source of morbidity and mortality in developing countries despite recent advances in aseptic techniques. There is no baseline information regarding SSI in our setting therefore it was necessary to conduct this study to establish the prevalence, pattern and predictors of surgical site infection at Bugando Medical Centre Mwanza (BMC), Tanzania.

**Methods:**

This was a cross-sectional prospective study involving all patients who underwent major surgery in surgical wards between July 2009 and March 2010. After informed written consent for the study and HIV testing, all patients who met inclusion criteria were consecutively enrolled into the study. Pre-operative, intra-operative and post operative data were collected using standardized data collection form. Wound specimens were collected and processed as per standard operative procedures; and susceptibility testing was done using disc diffusion technique. Data were analyzed using SPSS software version 15 and STATA.

**Results:**

Surgical site infection (SSI) was detected in 65 (26.0%) patients, of whom 56 (86.2%) and 9 (13.8%) had superficial and deep SSI respectively. Among 65 patients with clinical SSI, 56(86.2%) had positive aerobic culture. *Staphylococcus aureus *was the predominant organism 16/56 (28.6%); of which 3/16 (18.8%) were MRSA. This was followed by *Escherichia coli *14/56 (25%) and *Klebsiella pneumoniae *10/56 (17.9%). Among the *Escherichia coli *and *Klebsiella pneumoniae *isolates 9(64.3%) and 8(80%) were ESBL producers respectively. A total of 37/250 (14.8%) patients were HIV positive with a mean CD4 count of 296 cells/ml. Using multivariate logistic regression analysis, presence of pre-morbid illness (OR = 6.1), use of drain (OR = 15.3), use of iodine alone in skin preparation (OR = 17.6), duration of operation ≥ 3 hours (OR = 3.2) and cigarette smoking (OR = 9.6) significantly predicted surgical site infection (SSI)

**Conclusion:**

SSI is common among patients admitted in surgical wards at BMC and pre-morbid illness, use of drain, iodine alone in skin preparation, prolonged duration of the operation and cigarette smoking were found to predict SSI. Prevention strategies focusing on factors associated with SSI is necessary in order to reduce the rate of SSI in our setting.

## Background

Surgical site infections (SSIs) have been reported to be one of the most common causes of nosocomial infections; is accounting 20% to 25% of all nosocomial infections worldwide [1]. SSIs have been responsible for the increasing cost; morbidity and mortality related to surgical operations and continue to be a major problem worldwide [2]. Globally, surgical site infection rates have been reported to range from 2.5% to 41.9% [3-9]. In the United States, approximately 2% to 5% of the 16 million patients undergoing surgical procedures each year have postoperative surgical site infections [10].

In Tanzania, surgical site infections are still one of the leading causes of morbidity and mortality among patients undergoing major surgery. Previous studies conducted in a district and a tertiary hospital in Tanzania reported the surgical site infections rate of 24% and 19.4% respectively [11,12]. Surgical site infection is one of the most common preventable complications following major surgery at Bugando Medical Centre and represents a significant burden in terms of patient morbidity, mortality and hospital costs.

Despite improvements in operating room practices, instrument sterilization methods, better surgical technique and the best efforts of infection prevention strategies, surgical site infections remain a major cause of hospital-acquired infections and rates are increasing globally even in hospitals with most modern facilities and standard protocols of preoperative preparation and antibiotic prophylaxis. Moreover, in developing countries where resources are limited, even basic life-saving operations, such as appendectomies and cesarean sections, are associated with high infection rates and mortality [4,10].

This study was conducted to establish the prevalence, patterns and predictors of surgical site infection in our local setting. Here we document multiple factors predicting SSI in a large tertiary hospital in Mwanza, Northwestern, Tanzania.

## Methods

### Study design

This was an analytical cross-sectional prospective study among patients undergoing major surgery at BMC over a period of 9 months from July 2009 to March 2010. The study was conducted in the surgical wards of Bugando Medical Centre. Bugando Medical Centre (BMC) is a tertiary hospital with bed capacity of 900 and serves about 13 million people.

### Sampling

Sample size was obtained using Kish and Lisle method, using an incidence of 19.4% from a previous study and the infection rate among wound classes and the use of drain were also considered [12], the sample size obtained was 250. All patients of all age groups and gender undergoing major surgical procedures with visible incision (laparotomy, excisional biopsy, appendicectomy, thyroidectomy, herniotomy, mastectomy, amputations, open prostatectomy, cholecystectomy, thoracotomy, splenectomy etc) at Bugando Medical Centre who consented for the study were serially recruited until the sample size was reached. The study was approved by BMC/WBUCHS ethics review board.

### Data collection and Laboratory procedures

Predictor variables such as patient characteristics, preoperative data, intra-operative data and postoperative data were obtained using a standardized data collection form. All patients who underwent major surgery with visible incisions were eligible to participate in the study and requested to consent for study and HIV-testing before being enrolled into the study. HIV test was done using national algorithm of rapid test [13].

The patients were assessed preoperatively, intraoperatively and postoperatively. Details that were recorded included; type of surgery, wound class, type and duration of operation, antimicrobial prophylaxis, use of drain, preoperative hospital stay and total hospital stay. Each patient was followed up from the time of admission until time of the discharge and 30 days postoperatively. Surgical wound was inspected at the time of the first dressing and weekly thereafter for 30 days. Superficial surgical site infection was diagnosed if any one of the following criteria was fulfilled: purulent drainage from the superficial incision, organisms isolated from an aseptically obtained culture of fluid or tissue from the superficial incision, at least one of the following signs or symptoms of infection: pain or tenderness, localized swelling, redness, or heat, and superficial incision is deliberately opened by surgeon, and is culture-positive or not cultured. Deep surgical site infection was diagnosed if any one of the following criteria was fulfilled; purulent drainage from the deep incision but not from the organ/space component of the surgical site, a deep incision spontaneously dehisces or is deliberately opened by a surgeon and is culture-positive or not cultured and the patient has at least one of the following signs or symptoms: fever (> 38°C), or localized pain or tenderness. A culture-negative finding does not meet this criterion, an abscess or other evidence of infection involving the deep incision is found on direct examination, during reoperation, or by histopathological or radiological examination, diagnosis of a deep incisional SSI by a surgeon or attending physician. http://www.cdc.gov/nhsn/PDFs/pscManual/9pscSSIcurrent.pdf.

Pus or pus swabs were obtained from surgical incision and transported to the laboratory within an hour of collection. In the laboratory, the specimens were registered in the log books and processed as per standard operative procedures. Bacterial identification was done using an in house biochemical panel [14,15]. Antibacterial susceptibility testing to various antibiotics was performed using disc diffusion methods as previously described [16]. In addition, blood was taken from all patients for random blood sugar testing and CD4 enumeration in HIV positive status.

### Data analysis

Data were entered into a computer using SPSS software version 15 and analyzed using STATA software 10 according to the objectives of the study. Chi- square test was used to determine for the significance associations between the predictor and outcome variables to all categorical variables and odds ratios were calculated to test for the strength of association between predictor variables. In each variable reference variable was labeled as 1 in case of premorbidity each variable was compared to all others. Significance was defined as a p-value of less than 0.05. In addition to univariate analysis multivariate logistic regression analysis was performed.

### Study limitations

Failure to perform anaerobic culture might have contributed to the low prevalence of SSI due to inability to establish presence of organisms that require such environment (anaerobic bacteria). The absence of antibiotic policy in the surgical wards might have influenced the results of this study. Other surgical departments such orthopedic and gynecological departments were not included.

## Results

### Study population and demographic characteristics

A total number of 941 patients underwent major surgery at Bugando Medical Centre during the study period. Two-hundred sixty five patients fulfilled the inclusion criteria of these, 15 patients were excluded from the analysis due to loss of follow up Surgical site infection (SSI) was detected in 65 patients, giving an overall infection rate of 26.0% (table 1), of which 56 (86.2%) were superficial SSIs and 9(13.8%) were deep SSIs. Various procedures as seen in table 1 were done, laparotomy (61) due to various reasons such as peritonitis, intestinal obstruction, intestinal perforation was the leading procedure followed by open prostatectomy (26), excisional biopsy (23), thyroidectomy (21), appendicectomy (21) and others. The time taken for SSIs to develop ranged from 3-12 days with a mean of 6.23 days. The mean age was 38 years with standard deviation of 22.12 years. There were 116 (46.4%) males and 134 (53.6%) females (Table 2). SSI rate between male and female was 58% and 40% respectively (p-value = 0.01).

**Table 1:** Surgical procedures and SSI rate in each procedure

Surgical Procedure	SSI Positive	SSI negative	Total
Laparotomy	17 (27.9%)	44 (72.1%)	61
Open prostatectomy	8 (30.8%)	18 (69.8%)	26
Excisional Biopsy	7 (30.4%)	16 (69.4%)	23
Thyroidectomy	1 (4.8%)	20 (95.2%)	21
Appendicectomy	3 (15%)	17 (85%)	20
Herniotomy	0 (0.0%)	14(100%)	14
Spinal Bifida Repair	6 (46.2%)	7 (53.8%)	13
Mastectomy	5 (45.5%)	6 (55.5%)	11
Mayors Repair	3 (27.3%)	8 (62.7%)	11
Amputation	1 (12.5%)	7 (87.5%)	8
Cholecystectomy	1 (14.3%)	6 (85.7%)	7
Thoracotomy	4 (66.7%)	2 (33.3%)	6
Splenectomy	1 (33.3%)	2 (67.7%)	3
Cystolithotomy	1 (33.3%)	2 (67.7%)	3
Skull elevation	0 (0.0%)	3 (100%)	3
Others (20)	7 (35%)	13 (65%)	20

**Total**	**65 (26%)**	**185 (74%)**	**250**

Others (Bronchotomy (1), Cleft lip repair (2), Contracture release (1), corrective osteotomy (2), mamoplasty (1), penis amputation (2), pinnaplasty (2), Stripping (1), Urethroplasty (2), Varicolectomy (1), Z-plasty (2)

**Table 2:** Patients factors associated with Surgical Site Infection (SSI) at Bugando Medical Centre

Variable	SSI				
	**Yes n (%)** **N = 65**	**No n (%)** **N = 185**	**P value**	**OR**	**95% CI**

**Age in years**					
< 21	12 (18.5%)	49 (26.4%)		1	
21-40	18 (27.6%)	59 (32.9%)	0.60	1.24	0.54-2.83
41-60	21 (32.3%)	47 (25.4%)	0.15	1.82	0.81-4.11
> 61	14 (21.5%)	30 (16.2%)	0.16	1.91	0.78-4.66

**Sex**					
Female	26 (40%)	108 (58.3%)		1	
Male	39 (60%)	77 (42.7%)	0.01	2.12	1.19-3.70

**Pre-morbidity**					
None Diabetes	25(38.5%) 9 (13.8%)	167 (90.3) 1 (0.5%)	< 0.001 0.001	0.072 29.6	0.03-0.17 4.3-281
Hypertension	15(23.1%)	13 (7.0%)	0.002	4.0	2.1-10.8
HIV/AIDS	7 (10.7%)	2 (1.0%)	< 0.001	11.0	2.6-64.2

**Pre-morbidity**					
Absent	26 (40%)	168 (90.8%)		1	
Present	39 (60%)	17 (9.2%)	< 0.001	14.8	7.3-29.95

**Smoking**					
No	37 (56.9%)	180 (97.3%)		1	
Yes	28 (43.1%)	5 (2.7%)	< 0.001	27	9.87-75.2

### Predictors of SSI

#### Pre-morbid illness

Fifty-seven (22.8%) patients had pre-morbid illness namely diabetes Mellitus 18 (7.2%), hypertension 37 (14.8%) and HIV 37 (14.8%). The SSI rates for patients with pre-morbidity and those without were 70.2% and 38.4% respectively (p-value = 0.002) (Table 2). Mean CD 4 count was 296.35 cells/μl, the rate of SSI among HIV patients with CD count below 200 cells/μl and those with CD4 count of 200 cells/μl and above were 56.3% and 18, 8% respectively (*p*-value = 0.0001).

#### History of cigarette smoking

In this study, 33 (13.2%) patients had history of cigarette smoking. Of these 28 patients (84.8%) developed SSI. There was a statistically significant association between cigarette smoking and SSI (p-value = 0.001) as seen in Table 2.

#### Antimicrobial prophylaxis

Forty-one patients (16.4%) received preoperative antimicrobial prophylaxis. Majority of the patients 27(65%) received ceftriaxone and metronidazole; ampicloxacillin and gentamicin 3 (7.3%); ceftriaxone alone 3 (7.3%); ceftriaxone and gentamicin 3 (7.3%); cloxacillin 3 (7.3%) and ampicloxacillin and metronidazole 2 (4.9%). Antibiotics prophylaxis was administered in the following procedures; laparatomy (19), appendectomy (10), amputation (4), thoracotomy (2), mayo repairs (1), splenectomy (1), excisional biopsy (1), spinal bifida repair (1) and bronchotomy (1). The timing of preoperative antimicrobial prophylaxis ranged from 1 hour to 5 hours with the mean of 1.68 hours and standard deviation of 0.96 hour. The rate of SSI among patients who received pre-operative antimicrobial prophylaxis and those who did not receive were 14.63% and 28.23% respectively *(p*-value = 0.07) Table 3.

**Table 3:** Pre operative factors associated with surgical site infection at Bugando medical centre

Variable	Surgical site infection				
	**Yes n (%)**, **N = 65**	**No n (%)**, **N = 185**	**P value**	**OR**	**95% CI**

**Preoperative duration of hospitalization**					
≤ 7 days	41 (63%)	140 (75.7%)		1	
> 7 days	24 (37%)	45 (24.3%)	0.05	1.82	0.99-3.33

**Antimicrobial prophylaxis**					
Yes	6 (9.2%)	35 (18.9%)		1	
No	59 (90.8%)	150 (81.1%)	0.08	2.29	0.91-5.73

**Hair removal**					
Yes	1 (1.5%)	5 (2.7%)	0.59	1.78	0.20-15.5
No	64 (98.5%)	180 (97.3%)		1	

**ASA**					
I	30 (46.2%)	168 ((90.8%)		1	
II	27 (41.5%)	16 (8.6%)	< 0.001	9.45	4.55-16.6
III	8 (12.3%)	1 (0.6%)	< 0.001	44.8	5.4-371.3

**Skin preparation**					
Iodine alone	38 (58.5%)	16(8.7%)	< 0.001	13.9	6.8-28.2
Iodine and spirit	27 (41.5%)	158 (86.5%)		1	

ASA: American Society of Anesthesiologists

#### American Society of Anesthesiologists (ASA) classification, skin preparation, use of drain

The SSI rates for ASA classification I, II and III were 15.2%, 62.8% and 88.9% respectively *(p*-value = 0.001) Table 2. The majority of patients 185 (74.0%) used a combination of iodine and spirit for skin preparation. The SSI rates for iodine alone and Iodine + Spirit were 70.4% and 14.6%% respectively (p = 0.0001) Table 3. In this study, the use of drain was recorded in 54 patients (21.6%). SSI rates among patients who used drain and those who did not use were 48.2% and 19.9% respectively (p = 0.0001) Table 3.

#### Duration of operation

The duration of operation ranged from 40 minutes to 6 hours with a mean of 1.9 and standard deviation of 0.83. Mode and median were 2.0 and 2.0 hours respectively. The SSI rate in patient with duration of operation < 3 hours was 20.9% and 50% in those which had duration of operation ≥ 3 hours. There was statistically significant association between the duration of operation and SSI *(p*-value = 0.0001) (Table 3).

#### Post-operative antibiotic prophylaxis

All, except three patients who underwent excisional biopsy, were treated with antibiotics after the surgical operation. The frequency of post-operative antibiotics prescription were; ampicloxacillin (24.3%), ceftriaxone -metronidazole (23%), ceftriaxone (9.7%), ampicloxacillin -gentamicin (8.5%), ampicloxacillin -metronidazole (8%), gentamicin (7.2%), ceftriaxone-ampicloxacillin (6.1%), ciprofloxacin (4.8%), chloramphenicol (4.8%) ampicloxacillin-metronidazole-gentamicin (3.2%).

#### Surgical wound classification

Among the 250 patients, 193 (77.2%) had clean surgeries, 46 (18.4%) had clean-contaminated surgeries and 11 (4.4%) had contaminated surgeries. There were no cases of dirty surgery. The rate of SSI was 21.24%, 43.48% and 36.36% in clean, clean -contaminated and contaminated wounds respectively (Table 4).

**Table 4:** Intra operative factors associated with surgical site infection at Bugando medical centre

Variable	SSI				
	**Yes n (%)** **N = 65**	**No n (%)** **N = 185**	**P value**	**OR**	**95% CI**

**Nature of operation**					
Elective	54 (83%)	142 (76.7%)		1	
Emergency	11 (17%)	43 (23.3%)	0.29	0.67	0.32-1.39

**Type of anaesthesia**					
GA	56 (86.1%)	161 (87%)		1	
SAB	9 (13.9%)	24 (13%)	0.86	1.07	0.47-2.46

**Surgeon**					
Registrar	7 (10.7%)	32 (17.3%)		1	
Resident	13 (20%)	37 (20%)	0.37	1.6	0.57-4.5
Specialist	45 (69.2%)	116 (62,7%)	0.21	1.8	0.73-4.3

**Use of drain**					
Yes	26 (40%)	28 (15.1%)	< 0.001	3.8	2.0-7.14
No	39 (60%)	157 (84.9%)		1	

**Wound classification**					
Clean	41(63%)	152 (82%)		1	
Clean contaminated	20 (30.7%)	26 (14%)	0.002	2.85	1.44-5.61
Contaminated	4 (6.3%)	7 (4%)	0.25	2.12	0.59-7.59

**Suture**					
Nylon	44 (67.7%)	118 (63.8%)		1	
Silk	10 (15.4%)	9 (4.9%)	0.16	2.08	0.73-5.94
Vicryl	11 (16.9%)	57 (30.8%)	0.01	0.33	0.13-0.77

**Duration of operation**					
< 3 hours	43 (66.1%)	163 (88.1%)		1	
≥ 3 hours	22 (33.9%)	22 (11.9%)	< 0.001	3.8	1.92-7.48

**Number of people in theatre**					
≤ 6	11 (16.9%)	137 (74%)		1	
≥ 7	54 (83.1%)	48 (26%)	< 0.001	14	6.77-29

GA: General anesthesia, SAB: spinal anesthesia block, OR: Odd ratio, CI: confidence interval

#### Multivariate logistic regression

On multivariate logistic regression the following factors were significantly found to predict SSI; pre-morbid illness, duration of operation, use of iodine alone for skin preparation, use of drain and cigarette smoking (Table 5).

**Table 5:** Multivariate logistic regression analysis of factors associated with SSI

Predictor variable	Odd ratio	95% CI	P- value
Pre-morbid illness	6.1	1.3-28.9	0.022
ASA Classification above I	0.7	0.15-3.7	0.713
Use of drain	3.5	1.4-19.3	0.016
Use of iodine alone in skin preparation	17.6	6.5-48.1	< 0.001
Duration of Operation ≥ 3 hrs	3.2	1.1-9.3	0.033
Use of Vicryl	2.4	0.78-7.2	0.129
Cigarette smoking	9.6	2.4-38.3	0.001

#### Bacterial infection and susceptibility pattern

Out of 65 patients diagnosed to have SSI and specimens collected for microbiological investigation, 56 (86.2%) specimens from different patients had positive bacterial growth within 48 hours of incubation. Only five out of 56 cultured specimens (8.9%) had mixed growth. Common bacteria isolated after various procedures (table 1) were: *Staphylococcus aureus *16 (28.6%), *Escherichia coli *14 (25.0%) and *Klebsiella pneumoniae *10 (17.9%), while the least isolated bacteria was *Acinetobacter spp *1(1.8%). Nine (65%) and 8 (80%) of *Escherichia coli *and *Klebsiella pneumoniae *were found to be Extended Spectrum Beta -Lactamases (ESBL) producer respectively i.e resistant to first, second, third and fourth generation cephalosporins. Most of these ESBL producing isolates were isolated from the patients with laparotomy and open prostatectomy. MRSA was detected in 3(19%) of *Staphylococcus aureus*; of 3 MRSA isolates 2 were isolated from patients who had appendicectomy and one from corrective osteotomy. The resistance rates to ciprofloxacin were 86%, 80% and 54% for *Escherichia coli*, *Klebsiella pneumoniae *and *Staphylococcus aureus *respectively.

## Discussion

In this study, most of our patients were in the age group 31-40 and showed a female preponderance. Similar demographic observation was reported by another study in India [17]. The rate of SSI was significantly higher in male patients than in females. This could be explained by multiple risk factors in male such as cigarette smoking and HIV. Previous studies have shown that patients with pre-morbid illnesses, such as diabetes mellitus are at high risk of developing SSI due to their low immunity [18]; this was confirmed in this study. As described previously cigarette smoking was significantly found to be associated with SSI in the multivariate analysis [19]. Cigarette smoking has been reported to have an impact on wound healing through impairment of tissue oxygenation and local hypoxia via vasoconstriction [20].

The prevalence of HIV in this study was found to be higher than in general population this has been observed previously [21]. In the present study, the rate of SSI was found to be significantly higher in HIV positive patients than non HIV patients *(p*-value < 0.001). Also higher rate of SSI was observed among HIV patients with CD count below 200 cells/μl (*p*-value = 0.0001).

Prolonged pre-operative duration of hospitalization with exposure to hospital environment has been reported to increase the rate of surgical infection [22]. In this study a hospitalization of more than 7 days prior to surgery increased the risk of SSI by 2 fold. However in the present study, there were no significant differences in the rate of SSI between patients who received antibiotic prophylaxis and those who didn't. Despite lacking of significant association between preoperative antibiotics and SSI in this study, the authors still believe that antibiotic prophylaxis is most effective in preventing surgical site infections when administered 30 to 60 minutes before the start of surgery. The lack of significance could partly be explained by a non existing antibiotic policy regarding different procedures in these patients; only 16.4% of patients received pre-operative antibiotic prophylaxis. The findings of this study necessitate the introduction of evidence based antibiotic policy in this hospital and other hospitals in developing countries.

In this study in the univariate analysis it was observed that, the rate of SSI was significantly associated with ASA classification; similar observations have been made by other studies [23-25]. In this study ASA class 3 increased the risk for SSIs 45 times.

The number of people in theatre during operation has been considered as an independent predictor of SSI [26]. In the present study, the number of people more than 7 in theatre was significantly associated with increased risk of SSI by 14 times. Also the study observed that the use of povodine alone was found to be associated with higher SSI rates in both univariate and multivariate analysis than when used in combination with either chlorhexidine or alcohol related solutions. Povodine iodine has a shorter activity than chlorhexidine and it is inactivated by blood and serum protein [27,28]. In addition the use of surgical drain has been reported to be associated with an increased risk of SSI which was confirmed in this study [24,27,28].

In agreement with other studies [29], the present study found that a length of operation of more than 3 hours leads to 4 times higher risk for SSI. Increasing the length of procedure theoretically increases the susceptibility of the wound by increasing bacterial exposure and the extent of tissue trauma (more extensive surgical procedure) and decreasing the tissue level of the prophylactic antibiotic.

Surgical wound classification has long been established as an important predictor of the postoperative surgical site infections [12,30,31]. In our study as in previous studies the risk of SSI was statistically higher in contaminated wounds than in clean and clean contaminated wounds.

*Staphylococcus aureus *was the commonest isolate for the postoperative wound infections. This is consistent with reports from other studies [5,32]. It has been found that in clean surgical procedures, *Staphylococcus aureus *from the exogenous environment or the patient's skin flora is the usual pathogen, whereas, in other categories of surgical procedures, including clean-contaminated, contaminated and dirty, the polymicrobial flora closely resembling the normal endogenous microflora of the affected site is the most frequently isolated pathogens [32]. Here, *Staphylococcus aureus *was the most common isolate in patients who underwent excisional biopsy, thoracotomy, mastectomy and appendicectomy.

*Escherichia coli *and *Klebsiella pneumoniae *were the most common gram-negative bacteria and were predominantly isolated from in laparotomy and open prostatectomy; similar findings have been reported in other studies [32,33]. The study also found that most of the pathogens were multiply resistant to the commonly prescribed antibiotics such as ampicillin, amoxycillin-clavulanic acid, co-trimoxazole, tetracycline, penicillin, gentamicin, erythromycin, and ceftriaxone. Other authors in Nigeria and Kenya reported a similar antimicrobial susceptibility pattern [34,35]. These findings reflect the widespread and indiscriminate use of antibiotics, coupled with poor patient compliance and self-treatment without prescription among African patients. The majority of gram negative isolates were sensitive to meropenem while gram positive being sensitive to vancomycin and clindamycin; this could be explained by the fact that these antibiotics are relatively rare in the hospital and are more expensive so they are rarely misused.

## Conclusion

Surgical site infections are a major problem in the surgical wards at Bugando Medical Centre and its incidence is higher than that reported in developed countries. Multi-resistant *Staphylococcus aureus *followed by *Escherichia coli *and *Klebsiella pneumoniae *are common bacteria causing SSIs at BMC. Pre-morbidity, use of drain, use of iodine alone in skin preparation, duration of operation of more or equal 3 hours and cigarette smoking were significantly found to predict SSI at BMC on multivariate analysis. A better surveillance system for SSI with feedback of appropriate data to surgeons is highly recommended to reduce the SSI rate in developing countries.

## Competing interests

The authors declare that they have no competing interests.

## Authors' contributions

BM participated in collecting specimens, collecting clinical data and follow up of the patients, SEM participated in the design and execution of the work, performed, microbiological procedures, data analysis, interpretation of data and preparation of the manuscript, PLC participated in collecting clinical data and manuscript writing, CI interpretation of the data and manuscript writing and WM designed the study. All authors have read and approved the final manuscript.

## Pre-publication history

The pre-publication history for this paper can be accessed here:

http://www.biomedcentral.com/1471-2482/11/21/prepub
